# An Ambivalent Embrace: Service Needs and Gaps for Asian Immigrants in New Destinations

**Published:** 2017

**Authors:** John J. Chin

**Affiliations:** Hunter College, City University of New York

**Keywords:** Immigrants, New Destinations, Smaller Cities, Asian Americans, Health Care, Social Services, Cultural Competence

## Abstract

Asian immigrants to the U.S. are settling in “new destinations,” but there has been little research on their health care and social service needs. Our analysis of Census data to identify cities with the fastest Asian immigrant population growth (1990–2000) yielded 33 smaller cities in 13 states. The cities ranged in population from 7,677 to 86,660; were spread across 13 states in the Northeast, South, and Midwest regions of the US; and varied widely demographically. Pilot surveys conducted in 2009 indicated that, although many residents had positive attitudes towards immigrants, many were also concerned about job competition and dilution of American culture. Respondents reported a number of immigrant-targeted services but also service gaps and intergroup violence. We characterize smaller new destination cities’ mixed response to their fast-growing immigrant populations as an “ambivalent embrace.” Service gaps may be related to small city size and relatively small Asian immigrant population size, despite rapid population growth. Funding shortages were also cited as obstacles to cities’ responsiveness, suggesting the importance of state and federal government aid.

## INTRODUCTION

The incorporation of immigrants in the countries where they settle is an ongoing concern of immigration researchers and policy-makers because immigrants impact countries’ economic health, social and cultural life, and balance of political power. Policies and social attitudes regarding immigrants also both reflect and constitute fundamental aspects of communities’ and nations’ identities – conveying whether they embrace inclusion or prefer exclusion. Traditional immigrant gateways such as New York City and Los Angeles, with long histories of receiving large numbers of immigrants, are largely pro-immigrant and make concerted efforts to bridge language barriers, celebrate cultural differences and promote tolerance and non-discrimination through local policy, local government programs and public statements by local officials. However, many immigrants are now settling in what scholars have called “new destination” cities that have little experience with receiving immigrants. The term “new destination” denotes cities that immigrants have more recently chosen for settlement, in contrast to traditional immigrant gateway cities (e.g., New York City, Los Angeles, Chicago) that have a long history of immigrant settlement, dating back to the large immigrant influxes of the mid-nineteenth century.

Although variations exist, immigrants in traditional immigrant gateways have access to established immigrant institutions (such as ethnic banks and social service organizations) and benefit from local government initiatives to support newcomers. Less is known about the characteristics of immigrants in new destination cities and how these cities accommodate immigrants’ needs, and immigration scholars have called for new research that examines these more recent immigrant destinations (Marrow, 2005). There is a growing body of literature on the experiences of Latino immigrants in areas inexperienced with immigration (Gouveia & Stull, 1997; Guthey, 2001; Hernandez-Leon & Zuniga, 2000; Marrow, 2011; Massey, 2008). However, aside from the literature on Southeast Asian refugee settlement in the 1970s and 80s (Anderson, 1990; Rutledge, 1992), there are few accounts of Asian immigrants in new destinations (one is a study of Southeast Asian immigrants in meatpacking factories in Kansas (Benson, 1994)). Furthermore, the extent to which services targeted to Asian immigrants exist in new destination cities has not been examined systematically. Understanding the experiences of Asian immigrants is increasingly important as Asians now make up the largest racial group among new immigrants to the US (Pew Research Center, 2012).

This paper aims to fill a critical gap in the literature on Asian immigrants in new destinations by identifying the cities with the fastest growing Asian immigrant populations and reporting on a pilot survey that documented these cities’ resources and deficits with regard to meeting immigrants’ needs. We present survey results concerning community attitudes towards immigrants and the degree to which these new destination cities have been able to meet the needs of immigrants. For context, we also provide a brief demographic and socioeconomic profile of these cities based on US Census data. Although one might expect small new destination cities to be unwelcoming towards Asian immigrants, our research suggests that their response can be characterized more accurately as ambivalent.

### Immigrants in New Destinations in the United States

#### Changing Immigrant Settlement Patterns.

The US has seen record-high levels of foreign-born population in recent decades. Changes in immigrants’ settlement patterns have also occurred. Traditionally, immigrants to the US have settled primarily in central cities of major metropolitan areas. Since the 1980s, however, new immigrant gateway cities – marked by more recent rapid growth in foreign-born population – have emerged. Many of these new immigrant gateways are in the South (Wainer, 2004) and Southwest, including cities such as Atlanta, Charlotte, Dallas, Las Vegas and Salt Lake City (Frey, 1999; Singer, 2004). Between 1990 and 2000, the South had the largest increase in the number of foreign-born persons (88 percent), followed by the Midwest (65 percent), the West (50 percent) and the Northeast (38 percent) (Malone, Baluja, Costanzo, & Davis, 2003). Although most immigrants still reside in the largest immigrant-receiving cities, patterns of immigrant settlement have shifted to the point where the six states with the largest foreign-born populations (California, New York, Texas, Florida, New Jersey, and Illinois) saw their collective share of the nation’s immigrants decline in the 1990s for the first time in history, from 72.9 percent in 1990 to 68.5 percent in 2000 (Singer, 2004:2).

Settlement patterns in and around immigrant gateway cities have also been different in recent decades. Whereas immigrants tended to settle first in central cities, they are now heading directly to American suburbs, along with more established immigrants who have moved within the US from central cities to suburbs. This new pattern of settlement is observed in both new and traditional immigrant gateway cities. In the 45 traditional and emerging immigrant gateway metropolitan areas studied by Singer (2004:10), the percentage of foreign-born persons living in central city areas declined from 54 percent in 1970 to 43 percent in 2000.

The concept of a “suburban” migration may be slightly misleading in that it may suggest that immigrants are settling in relatively affluent areas within major metropolitan areas. This is often the case, but in many instances these “suburbs” are small cities and towns that are not linked to any major city. For instance, one of our study cities, the City of Salisbury, is isolated on Maryland’s Eastern Shore, with a 108-mile two-hour-plus drive on small highways separating it from Baltimore. Many of Salisbury’s immigrants, including a sizable number of Koreans, were drawn to the city to work in the area’s chicken processing factories (Rural Migration News, 2000). Immigrants in these small cities may have limited access to the kinds of resources available in large cities that have a longer history of receiving immigrants, and understanding these small new destination cities’ assets and challenges is important for understanding the changing immigrant experience in the US.

#### Assets and Challenges in New Destination Cities.

Unprecedented growth of foreign-born populations in new destination cities – all small cities in this case – may give rise to adjustment problems on both the receiving and entering sides. Small new destination cities may also have assets that facilitate effective incorporation of immigrant populations. Some of these assets are the qualities that attracted immigrants to these areas in the first place. For example, the lower cost of living in small cities, which appears to be a driving force for secondary migration (Fix, Zimmerman, & Passel, 2001; Holtzman, 2000), may allow for a better quality of life, particularly for lower-income immigrants. Small cities may also be more “livable,” particularly for immigrants originally from small towns and villages. Characteristics of some small cities, including a strong sense of community and concern for neighbors, can also facilitate a willingness to respond to immigrant needs.

Small city governments have the potential for greater adaptive capacity to respond to immigrant needs. In small cities, the citizenry is closer to the government and influencing government is easier; local governments can more easily monitor community needs; and decision-making authority is less fragmented, increasing the probability that decisions will be implemented (Detorres, 1985). Small city size has also been linked to greater levels of civic participation (Oliver, 2000). This asset of small city size holds regardless of the size of the surrounding metropolitan area (Oliver, 2000), which suggests that there may be commonalities in immigrants’ experiences across small cities, regardless of whether they are located in major metropolitan areas or are more isolated.

Small cities also have characteristics that may impede immigrant incorporation. The very qualities of small cities that improve government responsiveness to citizens’ demands and encourage civic participation may result in more virulent anti-immigrant initiatives if general community sentiment sways in that direction. Farmers’ Branch, Texas, for instance, one of our study cities, “passed an ordinance making English its official language, requiring landlords to check the immigration status of their tenants, … imposing fines of $500 per day on employers who hired undocumented workers,” and requiring police “to check the immigration status of persons held in custody” (Illinois Coalition for Immigrant and Refugee Rights, 2009).

Many small cities have been highly homogenous in ethnic composition since the early 1900s (Siegel & Waxman, 2001), and the rapid growth of post-1965 immigrant populations into visible minority groups may incite conflict and tension between US-born and immigrant populations in those areas (Frey, 1999; Steinberg & Baxter, 1998). In Lewiston, Maine, for instance, an influx of more than 1,000 Somali refugees into this overwhelmingly white city of fewer than 36,000 people prompted the mayor to write an open letter to Somali residents, printed in the local newspaper, asking them to dissuade other Somalis from settling there (Rabrenovic, 2007). Research on Vietnamese refugee resettlement found a positive statistical relationship between refugee personal adjustment and the proportion of the community that was neither black nor white, suggesting that communities that had a prior history with Latino or Asian populations would find the refugees to be less of an anomaly and therefore be more accepting of their presence (Starr & Roberts, 1982). This finding suggests that immigrants settling in small cities inexperienced with receiving immigrants may face more hostility. Anti-immigrant prejudice can increase when immigrants are in competition with the native-born population over scarce resources, such as jobs, affordable housing and service dollars (Quillian, 1995). Media portrayals of Asian immigrants and refugees as a highly successful model minority may also exacerbate native-born workers’ anxiety and resentment (Melnick, 1990).

Compared to immigrants in cities with longer histories of receiving immigrants, immigrants in new destination cities are likely to have more recently immigrated and thus tend to be less established and less likely to be bilingual in English. These factors may contribute to immigrants’ lack of political organization and representation in civic affairs. Human service organizations in these locations are less likely to have resources needed primarily by immigrants, such as trained language interpreters (Siegel & Waxman, 2001). In small cities in particular, if trained interpreters are available, they are more likely to be acquainted with clients, which might embarrass the client or raise concerns regarding confidentiality (Bouchard, 2001). Small cities may also lack the critical mass of immigrants and leaders to start immigrant-run community-based organizations that could respond to immigrant needs.

Some industries in new destination areas, such as the meat-packing industry, have made targeted efforts to recruit immigrants into dangerous and undesirable factory work (Lamphere, Stepick, & Grenier, 1994), leaving immigrants vulnerable to exploitation without the protection they might experience in an ethnic enclave in a traditional immigrant gateway city (Portes & Manning, 1986; Wilson & Portes, 1980; Zhou, 1992). As an alternative to factory work, many immigrant entrepreneurs in traditional immigrant gateway cities develop small businesses in economic niches (e.g. dry cleaning) where language barriers are less of an issue (Kim, 1987; Light & Bonacich, 1988; Portes & Manning, 1986; Portes & Stepick, 1993; Waldinger, 1989). However, as with the case of immigrant-run community-based organizations, small new destination cities may lack the critical mass of immigrant entrepreneurs and customers to support and protect development of immigrant small businesses.

## METHODS

### Identification of Cities with High Asian Immigrant Growth

We used US Census data packaged by Geolytics (GeoLytics, 2002a, 2002b) – which holds geographic boundaries constant between Censuses – to examine Asian/Pacific Islander (API) population growth between 1990 and 2000 in Census-defined “places,” which are legally incorporated towns and cities or their equivalents (US Census Bureau, 2001).

Using a relatively conservative, stringent measure of “high growth,” cities selected for further analysis were those that had 100 percent or greater growth in API population between 1990 and 2000 and also had 1,000 or more APIs in 2000 (to exclude high-growth cities that nevertheless still had very small API populations), and had API populations that were at least 80 percent foreign-born in 2000 (to ensure that the API population was primarily an immigrant population).^1^ These selection criteria yielded 33 cities, all under 90,000 in total population and with API populations that were primarily Asian, rather than Pacific Islander. For the 33 cities that met our selection criteria, we used Census data to describe sociodemographic and economic characteristics of the total population and the emerging Asian communities, including ethnicity, country of origin, socioeconomic status, and labor force participation.

### Pilot Survey

Survey procedures were reviewed and approved by the author’s institution’s Human Research Protections Program. Within each of the 33 selected cities, we attempted to interview one key government official and one key Asian immigrant community leader, identified through internet searches and by referrals from cold contacts, also located through internet searches. Each person was asked to participate in an interviewer-administered structured survey by telephone (n = 33 cities × 2 individuals = 66 surveys).^2^ The surveys, completed between January 2009 and June 2009, focused on Asian immigrants’ daily life issues and on institutional and community barriers to and facilitators of services aiming to meet the specific needs of Asian immigrants.

Because of the small sample size and the primary purpose of providing an overview of the cities’ experiences, we conducted primarily descriptive statistical analyses. More complex exploratory statistical analyses were conducted and were found to have limited power as a result of the small sample size. However, an independent samples t-test is included in the results section to begin to examine differences in Asian immigrant experiences by Asian population size; this analysis also helped to illustrate potential directions for future research with larger sample sizes. In order to conduct this analysis, the survey data were merged with data on city characteristics (e.g., population size) from the 2000 US Census.

## RESULTS

### Profile of Cities with the Highest Foreign-Born API Growth

Our selection criteria yielded 33 cities (see Table 1 below), all of them small (ranging in total population from 7,677 to 86,660), a reflection of lower baseline levels of foreign-born persons in the denominator when calculating growth but also of changing settlements patterns. The resulting cities came from three of the four main regions of the US (Northeast, South, Midwest) and from 13 states. Although four of the five cities in the US with the largest foreign-born API populations in 2000 were in California (Los Angeles, San Diego, San Jose, and San Francisco), no cities from the Western region of the US met our selection for inclusion in this analysis. The most highly represented states were New Jersey (with ten cities on the list) and Michigan (six cities).

For each of the 33 cities that met our selection criteria, Table 1 below shows total population, API population change from 1990 to 2000, total API population in 2000, the percentage of the API population that was foreign-born in 2000, and the percentage of the total population that was foreign-born (this figure includes *all* foreign-born persons). Numbers in bold and italic highlight the minimums and maximums in each column. Because of our selection criteria, all cities experienced at least 100 percent growth in API population. Some cities experienced much more rapid growth. Hamtramck, Michigan, for instance, saw a more than fifteen-fold increase in its API population.

Despite high API population growth, APIs made up a small percentage of the total population in many of the 33 cities in 2000 (the lowest being 1.5 percent in Lynchburg, VA; data not shown). In a few cities, however, API representation was much higher, as in Palisades Park, New Jersey, where APIs were 41 percent of the population.

The cities ranged widely in their economic health, with wide variation in per capita income, median contract rent and labor force participation (see Table 1). Dunwoody, Georgia, had the highest median household and per capita incomes. Hamtramck, Michigan, stood out in terms of having the lowest value for all of the economic measures examined, which may unfortunately have precipitated a loss of immigrant population between 2000 and 2009,^3^ despite having experienced the highest growth in API immigrant population among the study cities in the previous decade.

Because our selection criteria specifically selected for cities that had high API growth, foreign-born Asians were the largest foreign-born group in most of the cities (ranging from 2.3 percent to 62.3 percent of the total foreign-born population in each city). However, a number of cities had other dominant foreign-born populations (data not shown). For example, although Fountainbleau, Florida, had high API population growth, Asians made up only 2.3 percent of the foreign-born population there while immigrants from Latin America made up 95.8 percent of the foreign-born population.

Asian immigrants were predominantly South Asian in 15 cities, East Asian in 13 cities, and Southeast Asian in five cities (data not shown). A number of cities had fairly even shares of all three groups, such as in Merrifield (Virginia), Maryland Heights (Missouri), Madison Heights (Michigan), Hackensack (New Jersey), West Des Moines (Iowa), and Lynchburg (Virginia). This suggests that in many cases, the conditions that attracted foreign-born Asians appealed to more than one Asian ethnic group. Still, in a number of cities – such as Palisades Park (New Jersey), Forest Park (Georgia) and Hamtramck (Michigan) – Asian foreign-born persons were almost exclusively from one sub-group, reflecting the continuing influence of intra-ethnic chain migration and highly specific ethnic economic niches.

### Survey Results

#### Community Attitudes Regarding Immigrants.

**Figure 1.**
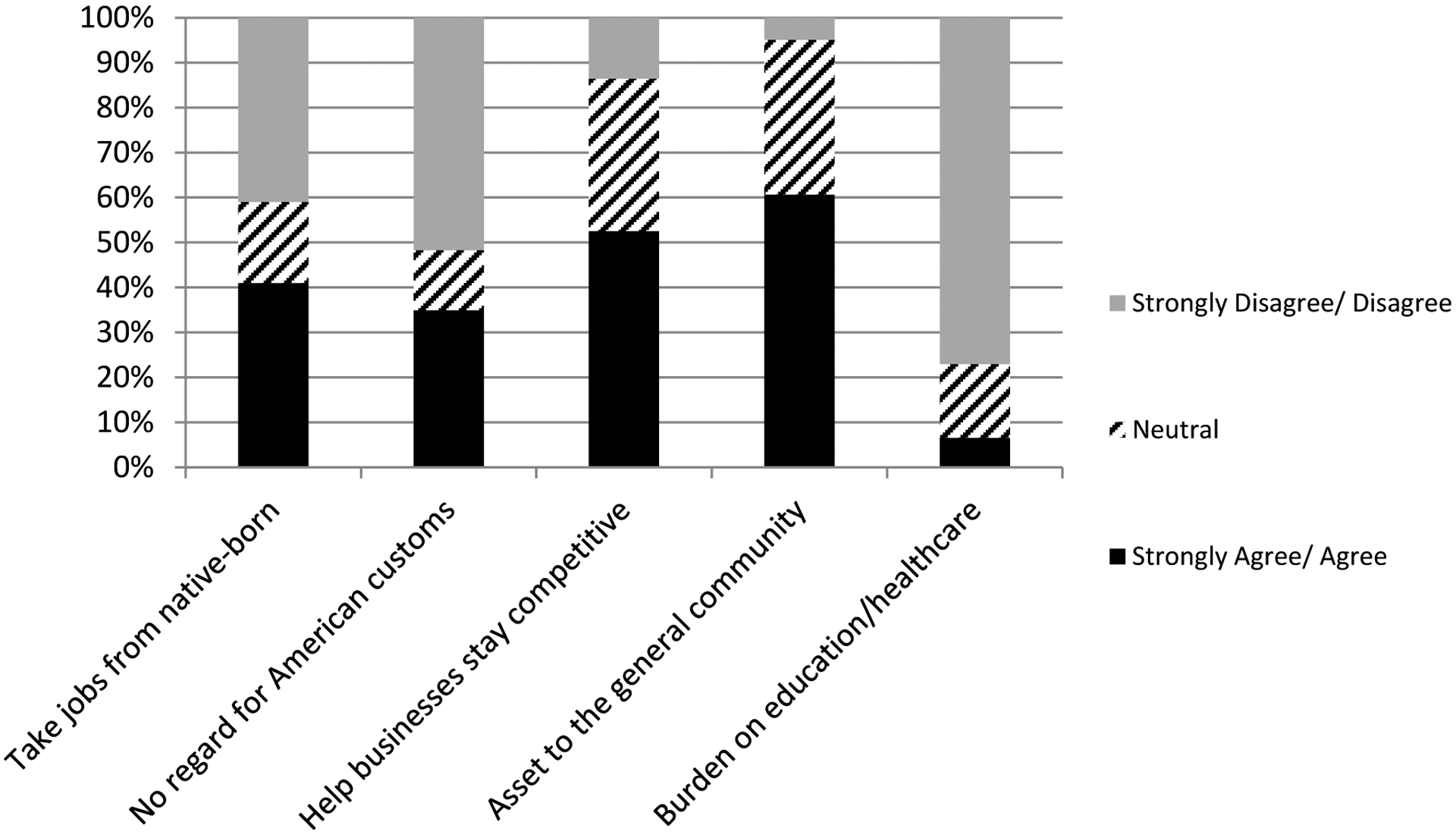
Respondents’ Perceptions of Community Attitudes Regarding Immigrants (n=59 to 61)

Telephone survey respondents in the 33 selected cities were asked about their perception of general community attitudes concerning their city’s largest Asian immigrant group (by country of origin), which we specifically identified for the respondents. Racially speaking, the “general community” for 22 out of the 33 cities in our study was mostly (more than 50 percent) white.^4^ For this section of the survey, respondents were asked whether they felt the general community would strongly agree, agree, feel neutral, disagree, or strongly disagree with a series of attitudinal statements. Respondents reported both negative and positive community sentiments towards Asian immigrants. For example, 41 percent of respondents said that community members would agree or strongly agree that Asian immigrants “have taken jobs that could go to native-born Americans” (see Figure 1), but an almost equal percentage of respondents said that community members would disagree with this statement. A substantial percentage of respondents (35 percent) said that community members would agree or strongly agree that Asian immigrants “do not have enough regard for American customs,” perhaps reflecting a discomfort with cultural differences or the possibility of cultural change as a result of immigrant influences; but a larger percentage of respondents said that community members would disagree with this statement.

Survey responses reflected more unequivocally pro-immigrant sentiments on some other questions. A majority of respondents said that community members would agree or strongly agree that Asian immigrants “have helped our businesses stay competitive” (53 percent) and “are an asset to the non-immigrant community” (61 percent), and, as importantly, very few respondents said that community members would disagree with these statements. Moreover, only about 7 percent said that their communities feel that immigrants “are a burden on our city’s education and health care system.” Survey results suggest that community members appreciate the economic boost that immigrants bring while remaining uneasy about potential job displacement and cultural change.

#### Intergroup Problems.

More than half of respondents noted that problems existed between the largest Asian immigrant group in their area and the non-immigrant community or other immigrant groups. For this section of the survey, respondents were presented with a list of problems and asked which of these had occurred in the previous five years; respondents were also invited to identify other problems not on the list. Thirty-one (31) out of 60 respondents (52 percent) reported that at least one type of problem had occurred in the last five years. In total, 114 problem types were noted by the 60 respondents (respondents could name more than one problem type). The most often-cited problem was ignorance of each others’ culture: almost half of the 60 respondents (47 percent) said this was a problem in the last five years, followed by ethnic tension (25 percent). More severe problems such as violence/assaults (23 percent) and discrimination (22 percent) were reported relatively frequently. The “other” category (10 percent) included vandalism of a Hindu temple and defacement of a storefront with the words “go home.”

Although the ability to conduct more complex statistical analyses was limited by the sample size, an independent samples t-test was conducted to begin to understand variations in experiences of Asian immigrants by city characteristics. Our analysis showed that in cities where respondents said that discrimination had been a problem in the last five years, the mean percentage of Asians/Pacific Islanders as a share of total city population was 13.8%, compared to 7.9% in cities where respondents said that discrimination had not been a problem (t=2.259, p=.028). This analysis suggests that Asian immigrants may experience more problems or backlash as their presence in a small city grows; at smaller shares of the total population, they may be seen as less threatening or intrusive.

Respondents were also asked to identify the types of actions taken in response to problems. Responses to problems included education, police intervention, outreach and counseling, task force creation, mediation, legal action, hiring of a Laotian police officer and a Chinese school teacher, creation of heritage week and “Asian Day” celebrations, and newspaper commentary on xenophobia. Community organizations were mentioned most frequently as initiators of solutions, followed by schools, unaffiliated individuals, police, and other government agencies.

#### Unmet Needs.

**Figure 2.**
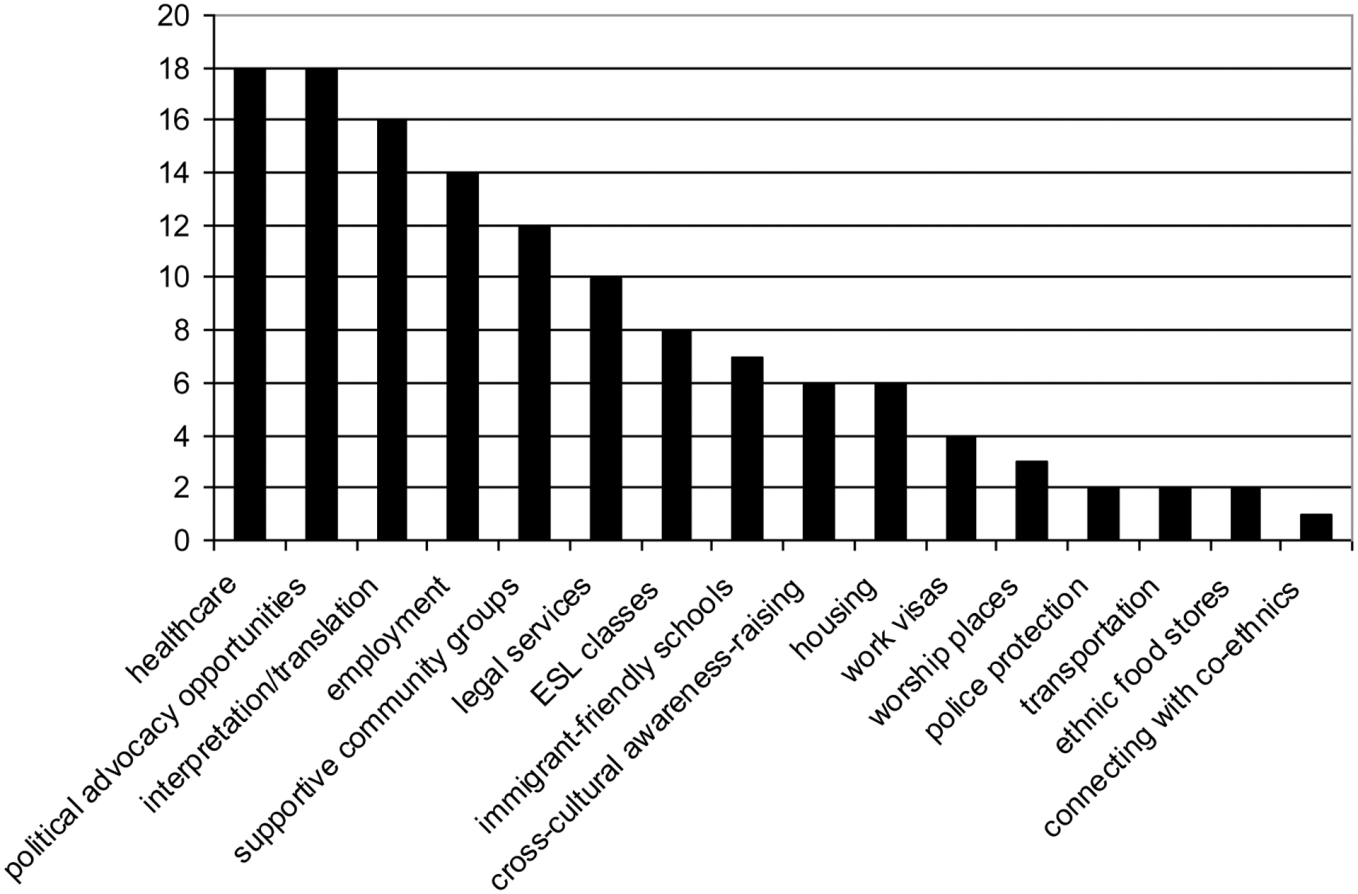
Top 3 Unmet Needs for Immigrants (n=50 respondents)

Respondents were provided with a list of 15 resources and services (including an “other” category) and then asked whether there was an unmet need for any of them among the foreign-born population in their city. The mean number of unmet needs identified per respondent (n=64) was 4.1 (SD=3.5). After respondents completed identifying unmet needs, they were asked to identify which were the top 3 unmet needs in rank order (50 respondents provided at least one ranked unmet need). Figure 2 below shows how often the various resources and services were mentioned as being among the top three unmet needs. Healthcare (n=18) and political advocacy opportunities (n=18) were ranked in the top three most often, followed closely by access to affordable language interpreters or translation services (n=16).

Respondents were given the opportunity to specify “other” unmet needs among the top three. These may be worth special mention since they were unprompted. In Figure 2 they are labeled and ranked as they were specified rather than grouped into one category as “other.” The most often mentioned “other” unmet need was cross-cultural awareness-raising (n=6).

#### Services Available for Immigrants.

**Figure 3.**
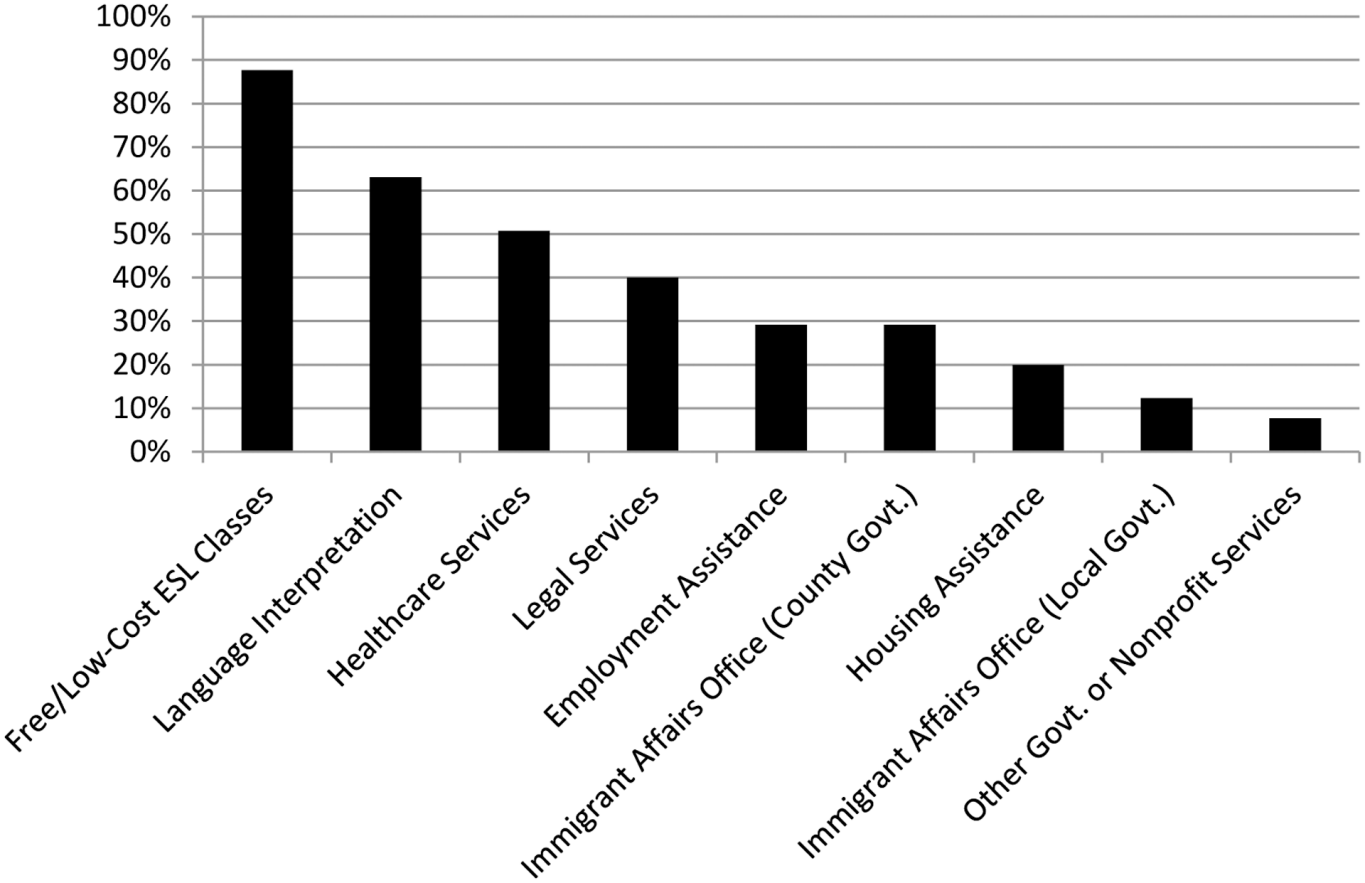
Percentage of Respondents Reporting the Availability of Government and Nonprofit Services for Immigrants (n=65)

We asked respondents to identify immigrant-targeted services available in their cities (see Figure 3 below), limiting the question to services provided by government and nonprofit organizations in order to exclude for-profit services, such as those provided by private immigration attorneys. The 65 respondents who answered this question identified a total of 221 services, for a mean of 3.4 services identified per respondent (SD=1.99). Most frequently mentioned by far were free/low-cost ESL classes, mentioned by 88 percent of respondents, followed by language interpretation services (63 percent), healthcare services (51 percent), legal services (40 percent) and employment assistance (29 percent). There seems, therefore, to be a fairly robust presence of immigrant-targeted services that parallel the first, third and fourth most often mentioned unmet needs (healthcare, interpretation/translation, and employment assistance respectively, as shown in Figure 2 above).

It appears that there are also efforts to improve immigrants’ access to county and local governments, addressing the need for “political advocacy opportunities” (the most often mentioned unmet need, along with healthcare). A number of respondents mentioned the presence of a county-level (29 percent) or city-level (12 percent) immigrant affairs office. Analyzing by city instead of respondent, 18 out of the 33 cities (55 percent) had either a county- or city-level immigrant affairs office according to at least one respondent per city. A review of governmental websites, however, suggests that respondents interpreted the term “immigrant affairs office” broadly. For instance, respondents in Scott Township, Pennsylvania, in Allegheny County, noted the existence of a county-level immigrant affairs office, but a review of Allegheny County’s website yielded only a web page devoted to providing information of interest to immigrants and to service providers working with immigrant populations (Allegheny County). The page is located in the Department of Human Services section of the county’s website, rather than in a section on community relations. Although Allegheny County has some immigrant-focused efforts, it seems to have no substantial “immigrant affairs office,” and the county’s efforts seem limited to the realm of service provision rather than political participation.

In contrast, Miami-Dade County, in which Doral is located, has an Office of Community Advocacy, which describes its purpose more broadly in terms of community relations and quality of life (Miami-Dade County, 2010), extending beyond the more limited objective of providing services to embrace larger ideas of social and cultural integration:

*“Miami-Dade County’s 2.5 million residents comprise a “melting pot” of various cultures and ideas. The Office of Community Advocacy (OCAd) was established to meet its unique community needs*…*[and to] promote productive community relations*…*for a better quality of life for all County residents.”*

*“*…*OCAd consists of five advisory boards: the Asian-American Advisory Board*…*, Black Affairs Advisory Board*…,…*Commission for Women*…*, Hispanic Affairs Advisory Board*…*, and the Community Relations Board*…*. Each board is charged with advocating for the special concerns of its constituents.”*

The case of Doral, Florida, demonstrates the potential benefits of sharing a county with a more established immigrant gateway city (i.e., Miami). Although respondents from both Scott Township and Doral noted the existence of a county-level immigrant affairs office, Miami-Dade County appears to be better positioned than Allegheny County to facilitate immigrants’ full integration into the political and cultural life of the community.

#### Materials Available in Immigrants’ Languages.

To further explore efforts to facilitate immigrants’ inclusion and participation in governmental affairs and to keep immigrant communities informed, we asked respondents whether important notices and educational information distributed by the local or county government are available in languages other than English. Of the 57 respondents who answered this question, a large majority (70 percent) said that non-English language notices and information were available. However, only 28 percent mentioned the availability of written informational materials in East, South and Southeast Asian languages in their cities, compared to 60 percent mentioning the availability of written materials in Spanish. This disparity is notable since our selection criteria favored cities with high foreign-born Asian population growth (of the 33 cities in the study, 61 percent had a majority or plurality of foreign-born persons from Asia among the total foreign-born population). The fact that Spanish speakers far outnumber speakers of Asian languages in the US overall may explain the wider availability of Spanish-language materials even in cities where Asian immigrants predominate. According to the 2000 Census, 28.1 million people aged 5 years and older spoke Spanish at home in the US, compared to 7 million people speaking an Asian or Pacific Islander language (Shin & Bruno, 2003:Figure 2).

#### Obstacles to Improving Local Governments’ Response to Immigrant Populations.

Our survey included an open-ended question on obstacles to improving local governments’ response to immigrant populations. Forty-three (43) of the 66 respondents provided an answer to this question (respondents could identify as many obstacles as they wished). The obstacles mentioned were organized into 10 categories (see Table 2 below). Many of the categories are overlapping. For example, obstacles related to cost and lack of funding, the most frequently mentioned obstacle (noted by 35 percent of respondents) overlaps with the low prioritization of immigrants’ needs, the second most often mentioned obstacle (28 percent). Also overlapping with these are obstacles related to a poor local economy, mentioned by 7 percent of respondents. These respondents suggested that competition for jobs and resources in a poor economy was a primary source of anti-immigrant sentiment and of lack of political will for helping immigrants.

Interestingly, a number of respondents (16 percent) noted obstacles rooted in the behaviors or attitudes of the immigrants themselves (categorized under “immigrants’ self-isolation”). Obstacles included in this category were immigrants’ “insular” cultures, limited English proficiency, undocumented immigrants’ fear of involvement in civic affairs, immigrants’ focus on work and school at the expense of civic engagement, and immigrants’ reluctance to ask for help. Further isolating immigrants was the inability to identify qualified interpreters and ESL teachers speaking needed languages, mentioned by 7 percent of respondents.

The “other” category (9 percent) includes local government offices’ lack of evening hours, difficulty in prioritizing which of the many languages to target for services (perhaps a problem that could be remedied with data), a general lack of civic engagement in the community overall, and lack of unification and political power in immigrant communities. Six of the 43 respondents to this question (14 percent) explicitly stated that there were no obstacles to improving local government’s response to immigrants.

## DISCUSSION

Immigrant settlement patterns in the US are changing, and many of the places with emerging immigrant populations are small cities that are relatively inexperienced with receiving immigrants. The cities with the fastest growing API foreign-born populations between 1990 and 2000 were all small (less than 90,000 in total population) and were spread across 13 states in the Northeast, South and Midwest regions of the US. Although small new destination cities that have limited experience with immigrants might be expected to hold negative views of immigrants, our survey uncovered a mix of sentiments, both positive and negative. Survey respondents reported intergroup tensions and violence, lack of translated materials and unmet needs, but also creative responses to problems (including educational programs, cultural celebrations, community forums, and hiring of Asian immigrant staff to key positions) and also a wide range of immigrant-targeted services. We have characterized new destination cities’ response to rapidly growing immigrant populations as an “ambivalent embrace” to suggest that these cities have been welcoming of new immigrants but also sometimes cautious or negative in their response. As indicated by our analysis of discrimination and Asian/Pacific Islander population share, small cities’ responses may become increasingly negative as immigrants’ presence in the city increases.

The prominence of political advocacy opportunities and cross-cultural awareness-raising as unmet needs indicates the importance of addressing not just economic integration and service needs, but also more directly attending to the political and social/cultural ramifications of immigrant population growth in smaller cities. A number of respondents mentioned the existence of city- or county-level immigrant affairs offices – which might facilitate immigrants’ access to local government, advocate on behalf of immigrants, and intervene when problems of intergroup relations arise – but some of these offices appear to have little substance and a narrow scope. Some of these gaps might be filled by private foundation or government grants that provide support to immigrant-led organizations and other community organizations, which can facilitate immigrants’ civic engagement and lead community awareness-raising efforts.

For most immigrant groups, overcoming language barriers is a fundamental step in successful incorporation into the civic, political and economic life of a place; however, only 28 percent of respondents said that materials were available in East, South or Southeast Asian languages despite the fact that 61 percent of the 33 cities had a majority or plurality of foreign-born persons from these groups among the total foreign-born population. Even when an Asian foreign-born population is substantial and growing, the wide variety of Asian languages spoken might make it harder to achieve a critical mass of speakers of any one Asian language to prompt the development of written materials in that language, especially in a small city where absolute population levels are low. Several respondents also mentioned that finding qualified bilingual interpreters for real-time interpretation was difficult, a problem that is likely to be greater in small cities. The power of critical mass to spur a governmental response to immigrants’ needs is demonstrated in New York City, where, for instance, in 2011 the Board of Elections mailed voting materials in English, Spanish, Chinese and Korean to all registered voters (NYC Board of Elections, 2011). To the extent that some translated materials can apply to multiple contexts, smaller cities may be able to use the translated written materials produced by larger cities that have substantial immigrant populations, for example, to convey preventive health information. Telephone-based real-time language interpretation can also help to fill gaps in face-to-face language interpretation or bilingual providers. Studies have documented the effectiveness of remote simultaneous medical interpreting (RSMI), where trained off-site interpreters provide simultaneous interpretation through wireless headsets worn by both the patient and the provider (Gany, 2007).

The new destination cities we studied offered many services, and yet unmet needs remained. Obstacles to improving the local government’s response to immigrants’ unmet needs included cost and lack of funding, low prioritization of immigrants’ needs, and immigrants’ self-isolation from civic affairs – which might be a form of “blaming the victim,” especially in the case of undocumented immigrants. Funding concerns and low prioritization of immigrants’ needs – by far the most often cited obstacles – are likely to be exacerbated by poor local economies, which tighten city budgets and fuel resentment towards immigrants, especially in cities that are disproportionately burdened by immigrant settlement but have little experience with receiving immigrants. Finding ways to leverage more aid from state and federal governments for these cities might allow them to prioritize initiatives that address immigrants’ needs.

Self-isolation of immigrants as an obstacle, coupled with a noted lack of political advocacy opportunities for immigrants, suggests that there may be untapped potential within immigrant communities for addressing unmet needs through community organizing and developing immigrant-led nonprofit community organizations, although establishing the critical mass needed for such developments will continue to be a challenge. In the meantime, immigrant communities may need to rely on nonprofit organizations that are not necessarily immigrant-initiated or led, especially in light of the lack of substantive immigrant affairs offices at the local or county governmental levels. Nonprofit community organizations figured prominently in survey results as responders to intergroup problems and as service providers. In small cities with potentially fewer public resources for immigrants and lacking the immigrant population base for the development of immigrant-led organizations, more generally targeted community organizations may play an important role in meeting immigrants’ needs and facilitating immigrants’ incorporation into community life and the public sphere.

Our survey method has several limitations. Survey respondents were chosen purposively rather than randomly, and only two respondents were interviewed in each city. The survey results, therefore, are not necessarily generalizable or representative of the views in those cities. Survey respondents were also asked in some cases for their perceptions of community views and also asked to report largely from memory on unmet needs and services available for immigrants. Their responses therefore may be biased. Still, using the telephone survey method with a small sample allowed us to gather data efficiently on all of the geographically dispersed cities that met our selection criteria, providing a comprehensive overview of the experiences of US cities with the fastest growing API immigrant populations between 1990 and 2000.

## CONCLUSION

A variety of future research projects may be helpful as next steps. A comparative case study that compares several different types of new destination cities and examines each city in more depth may help to illuminate the root causes of barriers and the facilitators of solutions. A participatory-based study that involves immigrant community members would provide a more direct understanding of immigrants’ experiences from their perspective. A new study that captures more recent dynamics would allow for understanding changes since our 2009 survey. Finally, a study with a larger sample size would provide more power for complex statistical modeling.

## Figures and Tables

**Table 1.** Demographic Characteristics & Economic Indicators for US Cities with Highest Growth of API Foreign-Born Pop., 1990–2000*

Place Name	Total Pop. in 2000	API % Change 1990–2000	Total API in 2000	% Foreign-Born among API	% Foreign-Born among Total Pop.	Per Capita Income	Median Contract Rent	2000 Labor Force Participation (%)
NJ, Edgewater	***7,677***	358.0	1,759	85.7	35.5	$42,650	***$1,158***	72.8
MI, Farmington	10,423	605.3	1,065	87.0	16.1	$32,452	$690	62.8
NJ, Little Ferry	10,800	163.2	1,845	***80.3***	31.1	$24,210	$768	68.1
VA, Merrifield	11,080	150.2	3,180	83.0	41.8	$32,819	$962	72.5
GA, Riverdale	12,453	118.1	1,049	81.7	15.2	$15,377	$568	70.3
NJ, Highland Park	13,999	199.5	1,926	86.6	29.2	$28,767	$802	69.5
NJ, Harrison	14,424	119.9	1,716	82.7	56.0	$18,490	$639	62.0
NJ, Palisades Park	17,073	141.1	***7,015***	83.1	57.0	$22,607	$851	63.3
PA, Scott Township	17,288	305.7	1,006	***88.4***	8.9	$24,439	$571	60.8
NJ, Avenel	17,552	239.4	3,296	82.2	25.9	$19,794	$794	57.0
FL, Doral	20,513	392.1	1,186	85.2	62.6	$27,705	$870	66.1
GA, Forest Park	21,293	179.8	1,320	82.4	21.4	$14,932	$510	62.1
MI, Hamtramck	22,976	***1441.2***	2,358	82.4	41.1	***$12,691***	***$378***	***49.9***
MD, Salisbury	24,159	230.6	***1,005***	80.8	6.7	$15,228	$463	63.3
MO, Maryland Heights	25,937	179.3	1,846	84.7	10.2	$24,918	$588	74.2
TX, Farmers Branch	28,325	***103.1***	1,029	80.8	25.2	$24,921	$678	69.2
MA, Norwood	28,587	243.4	1,384	81.2	11.8	$27,720	$820	66.8
MD, Pikesville	28,936	197.1	1,019	83.1	20.1	$41,035	$710	63.1
MI, Inkster	30,115	457.3	1,129	81.5	5.4	$16,711	$449	60.2
MI, Madison Heights	31,101	108.4	1,609	81.4	14.4	$21,429	$548	65.2
GA, Dunwoody	32,808	168.8	2,483	83.1	14.9	***$43,523***	$901	69.2
NJ, Pennsauken	35,703	206.3	1,556	81.7	7.9	$19,004	$531	62.7
MA, Woburn	37,258	308.8	1,826	82.5	9.6	$26,207	$809	69.2
NJ, Sayreville	40,377	275.9	4,184	81.0	20.1	$24,736	$713	65.5
NJ, Atlantic City	40,517	174.5	4,089	83.5	24.7	$15,402	$502	56.8
NJ, Hackensack	42,677	146.7	3,197	82.1	33.9	$26,856	$790	66.0
IA, West Des Moines	46,300	242.4	1,236	82.9	5.4	$31,405	$601	***77.5***
CT, Milford City	50,602	310.6	1,369	81.1	8.1	$28,773	$766	70.1
FL, Fountainbleau	59,518	169.3	1,139	83.9	***73.1***	$14,716	$705	57.9
VA, Lynchburg	65,269	126.0	1,001	82.5	***3.2***	$18,263	$383	59.8
OK, Edmond	68,514	124.7	2,126	81.9	5.2	$26,517	$461	70.0
MI, Wyoming	69,366	127.0	2,008	***80.3***	7.9	$19,287	$516	73.7
MI, Westland	86,660	217.3	2,386	84.4	6.8	$22,615	$574	67.0

* NOTE: Selection criteria included: 100 percent or greater increase in API population between 1990 and 2000, more than 1,000 APIs in 2000, and API population at least 80 percent foreign-born in 2000.

**Table 2.** Obstacles to Improving Local Governments’ Response to Immigrant Population Needs (n=43, respondents permitted to name more than one obstacle)

Obstacle	Percentage of Respondents who Mentioned Obstacle
Cost/Lack of Funding	35%
Low Priority	28%
Immigrants’ Self-Isolation	16%
None	14%
Other	9%
Low Awareness	9%
Unresponsive Local Government	9%
Poor Economy	7%
Lack of Qualified Bilingual Staff	7%
Anti-Immigrant/Undocumented Sentiment	5%

## Footnotes

1 We examined growth in API population as opposed to growth in API foreign-born population and then set the 80 percent foreign-born criterion for 2000 because comparable data on the API foreign-born population were not available for the 1990 and 2000 Censuses. Geolytics data for the 2000 Census included foreign-born status by race, whereas the 1990 data not include this information.

2 As planned, two interviews were conducted per city, but because of difficulties with recruitment, we sometimes interviewed non-immigrant civic leaders (such as the head of the local Chamber of Commerce) in place of government officials and in some cities interviewed two government officials/civic leaders or two ethnic community leaders – rather than interviewing one of each. To diagnose the potential impact of this recruitment problem, we ran statistical tests comparing government officials/civic leaders and ethnic community leaders – as well as non-Asian and Asian respondents – on some key questions and found few relevant differences in their views. Since we found few differences between types of respondents, and given the small sample size, we present only descriptive statistics with all respondent types combined. It should also be noted that we prioritized recruitment of health experts among government officials because one part of the study (not reported here) focused particularly on immigrants’ healthcare access. As a result, a sizeable number of respondents worked for health departments or other healthcare organizations (n=20). This may have resulted in a bias towards viewing healthcare as an important concern.

3 Our review of 2009 5-year American Community Survey (ACS) data showed that, of the 33 cities in our study, only three experienced a decline in foreign-born population between 2000 and 2009. Of these, the decline in Hamtramck (Michigan) was most severe at 6 percent, compared to 0.2 percent for Westland (Michigan) and 1.3 percent for Farmers Branch (Texas). For the remaining cities, increases in foreign-born population in the same period ranged from a low of 0.03 percent (Pikesville, Maryland) to 75.8 percent (West Des Moines, Iowa). Although the ACS data are not entirely comparable to the Census data from Geolytics that we have relied on for this paper, the ACS data can help to understand the direction of trends.

4 In an additional four cities, whites made up the largest population share without constituting a majority. Latinos constituted a majority in two cities as did African Americans. In the three remaining cities where there was no majority, APIs made up the largest share in one city, and African Americans made up the largest share in two cities.
